# Fournier’s gangrene of the penis caused by *Streptococcus dysgalactiae* subspecies *equisimilis:* case report and incidence study in a tertiary-care hospital

**DOI:** 10.1186/1471-2334-13-381

**Published:** 2013-08-20

**Authors:** Ram V Anantha, Katherine J Kasper, Kelcey G Patterson, Joseph J Zeppa, Johan Delport, John K McCormick

**Affiliations:** 1 Western University, London, Ontario, Canada; 2 Department of Microbiology and Immunology, Siebens-Drake Research Institute Room 133, Western University, 1400 Western Road, London N6G 2V4, Canada

**Keywords:** *Streptococcus dysgalactiae* subsp. *equisimilis*, Fournier’s gangrene, MALDI-TOF MS, Species identification

## Abstract

**Background:**

Fournier’s gangrene is a rare necrotizing soft tissue infection of the scrotum and penis. We report, to our knowledge, the first case of Fournier’s gangrene caused by *Streptococcus dysgalactiae* subsp*. equisimilis* (SDSE), a strain of pyogenic β-hemolytic streptococci that is increasingly being recognized as an important human pathogen.

**Case presentation:**

We describe a healthy 59 year-old Caucasian male who presented to the emergency department with Fournier’s gangrene of the penis and scrotum, with extension to the anterior abdominal wall. He underwent urgent surgical debridement of his scrotum, penis, and anterior abdomen. Swabs from the scrotum grew Gram-positive cocci, which were initially identified as *Streptococcus anginosus* group by matrix-assisted laser desorption ionization–time of flight mass spectrometry (MALDI-TOF MS). However, polymerase chain reaction (PCR) amplification and sequencing of the 16S rRNA gene identified the isolate as *Streptococcus dysgalatiae* subspecies *equisimilis* (SDSE). The incidences of invasive *S. anginosus* group and SDSE infections at the London Health Sciences Centre, a tertiary-care institution in southwestern Ontario, were determined between August 1, 2011 and August 31, 2012, revealing a slightly lower rate of SDSE (3.2 cases per 100,000 population) than other studies.

**Conclusions:**

This case highlights a unique disease manifestation of the emerging human pathogen *Streptococcus dysgalatiae* subspecies *equisimilis* that has not been previously reported. This case also underscores the limitations of MALDI-TOF MS in differentiating between closely-related streptococcal species which may have different pathogenic profiles.

## Background

Fournier’s gangrene is a rare necrotizing infection of the male genitalia [1,2]. It is classically characterized by intense pain and tenderness in the genitals, rapidly progressing to gangrene and septic shock. Risk factors include diabetes [1], immune compromise, drug use, obesity, and trauma to the perineum [2,3]. Most cases of Fournier’s gangrene are polymicrobial [2], and commonly isolated microorganisms include *Escherichia*, *Klebsiella*, *Bacteroides*, *Clostridium*, streptococci and enterococci [1]. Early therapy is critical, including surgical debridement, broad-spectrum antibiotics, and skin grafting [1-3]. We describe a case of Fournier’s gangrene in a healthy male caused by *Streptococcus dysgalactiae* subsp*. equisimilis* (SDSE), initially misidentified as *Streptococcus anginosus* group. SDSE is a pyogenic β-hemolytic *Streptococcus* that is emerging as a human pathogen with a similar disease profile to *S. pyogenes*[4-6]. While it primarily presents as skin and soft-tissue infections, including cellulitis and necrotizing fasciitis [4], SDSE can also cause endocarditis, rheumatic fever, and streptococcal toxic shock-like syndrome [5,6]. With an ever-increasing clinical burden, there is a need to accurately identify invasive SDSE infections.

## Case presentation

A 59 year-old, previously healthy Caucasian male presented with scrotal and penile pain for six days, and brownish-black discoloration of the scrotum. His past medical and surgical history was non-contributory and he did not take any medications. There was no history of trauma or sepsis in the genital area, and there were no symptoms of dysuria or hematuria. A review of systems was unremarkable. On examination, the patient was alert and oriented, but appeared unwell. He was also febrile (38.3°C), tachycardic (heart rate 116/min), and normotensive (blood pressure 136/79). His lower abdomen was erythematous with palpable subcutaneous emphysema extending to the umbilicus. His penile shaft was swollen and tender, and his scrotum was necrotic. Bloodwork revealed a white cell count of 17 × 10^9^/L, hyponatremia (125 mmol/L), hypochloremia (86 mmol/L), and elevated serum lactate (3.1 mmol/L). The patient’s international normalized ratio (INR) was 1.6, and his serum alanine-aminotransferase (ALT), and serum aspartate-aminotransferase (AST) were elevated at 127 U/L, and 66 U/L respectively. Blood glucose and serum creatinine were within normal limits.

Intravenous treatment with vancomycin (1 g every 12 h), and piperacillin-tazobactam (4.5 g every 8 h) was initiated. The patient was urgently taken to the operating room and underwent extensive debridement of his scrotum, penile shaft, and anterior abdomen, while preserving the testes and abdominal muscles. A small abscess cavity communicating with the necrotizing infection was identified in the right buttock and debrided. A suprapubic catheter was placed for urinary diversion, and the patient was admitted to the intensive care unit. After 48 hours, he underwent a transverse colostomy to divert stool from his perineum, and skin grafting to close his anterior abdominal wounds and penile shaft. His testes were tunnelled into his thighs, and after sixteen days in hospital, he was discharged home.

A swab from the scrotal tissue was taken during the initial operation, and was streaked on blood agar plates, resulting in a monoculture of uniformly-sized beta-haemolytic colonies. From this, the bacteria was isolated and subjected to matrix-assisted laser desorption ionization–time of flight mass spectrometry (MALDI-TOF MS) to identify the organism as *Streptococcus anginosus* group (score = 2.3), although the software (Biotyper software version 3.0) was unable to distinguish the species. Susceptibility testing was performed by the Kirby-Bauer disc diffusion method [7], and the organism was sensitive to ceftriaxone, clindamycin, erythromycin, penicillin, and vancomycin. Polymerase chain reaction (PCR) amplification (Table 1) and sequencing of the 16S rRNA amplicon from DNA isolated from overnight cultures of two isolated colonies identified the isolate as *Streptococcus dysgalatiae* subspecies *equisimilis*. This was further confirmed by sequencing the *emm* amplicon and performing a BLAST search on the Centers for Disease Control (CDC) streptococcal *emm* sequence database [8]: the isolate was identified as group G *Streptococcus emm* type *stG643.0*. Further PCR amplification experiments (Additional file 1: Figure S1) did not detect any of the 11 known streptococcal superantigen genes [9], when compared to genomic DNA preparations of *S. pyogenes* serotypes MGAS5005 [9], SF370 [10], MGAS8232 [10], and MGAS315 [10], which served as positive controls. In addition, proliferation assays [11] confirmed the absence of Group A streptococcal superantigen activity in the isolate (Addition file 1: Figure S2). Briefly, supernatants from overnight cultures of the isolate were added to fresh, gradient-purified human peripheral blood mononuclear cells (PBMCs; 2.0 × 10^5^ cells/well) in 96-well plates. After 3 days, ^3^H-thymidine was added for 18 h to measure proliferation. Supernatant from *S. pyogenes* strain MGAS5005 [9] served as a positive control.

**Table 1:** **List of primers used to amplify streptococcal superantigen genes, 16S ribosomal RNA (rRNA), and *emm *genes**

**Name**	**Sequence 5′-3′**	**Source**
SpeA forward	AAAGTTGCCATCTCTTGGTTC	Sigma genosys
SpeA reverse	CAAGAGGTATTTGCTCAACAAGAC	Sigma genosys
SpeC forward	TTTGAGCAGGCGTAATTCCT	Sigma genosys
SpeC reverse	TTCAACGACACACACATTAAACA	Sigma genosys
SpeG forward	ACCCCATGCGATTATGAAAA	Sigma genosys
SpeG reverse	GGGAGACCAAAAACATCGAC	Sigma genosys
SpeH forward	ATTCCAATGTTGTTCAAGCAAA	Sigma genosys
SpeH reverse	TGAGCGGTTACTTTCGGTTT	Sigma genosys
SpeI forward	TCCGCCATTTTCAGGTAGTT	Sigma genosys
SpeI reverse	TTTCCTTCCTCAAAGCCAGA	Sigma genosys
SpeJ forward	GCTCTCGACCTCAGAATCAA	Sigma genosys
SpeJ reverse	CTTTCATGGGTACGGAAGTG	Sigma genosys
SpeK forward	CAAACAAGGAACGCAATTGAT	Sigma genosys
SpeK reverse	GTGTCTAATGCCACCGTCT	Sigma genosys
SpeL forward	ATAAGTCAGCACCTTCCTCTTTC	Sigma genosys
SpeL reverse	AAATCTCCCGTTACCTTCCA	Sigma genosys
SpeM forward	AACTTCTTCTTCCTTAAAGCGTCT	Sigma genosys
SpeM reverse	TGCTGTGTTGGTTAATAGCGA	Sigma genosys
SmeZ forward	TTTCTCGTCCTGTGATTGGA	Sigma genosys
SmeZ reverse	AATGGGACGGAGAACATAGC	Sigma genosys
SSA forward	ACAGGTCAGCTTTTACAGCA	Sigma genosys
SSA reverse	GGGCATCATATCGTACCAAA	Sigma genosys
16S rRNA forward	AGAGTTTGATCCTGGCTCAG	Invitrogen life technologies
16S rRNA reverse	AAGGAGGTGATCCAGCCGCA	Invitrogen life technologies
*emm* genotyping primer forward	TATTCGCTTAGAAAATTAA	Invitrogen life technologies
*emm* genotyping primer reverse	GCAAGTTCTTCAGCTTGTTT	Invitrogen life technologies

We also used the London Health Sciences Centre (LHSC) microbiology database to retrospectively review patients with invasive *S. anginosus* group or SDSE infections at LHSC (a tertiary-care centre in Southwestern Ontario that serves a regional population of 435 000) between August 1, 2011 and August 31, 2012. The review was conducted according to the Helsinki Declaration, and approved by the Research Ethics Board of Western University (approval number 103036). Incidences were calculated using Statistics Canada census data from 2011 [12]. We identified 17 cases of invasive *S. anginosus* group infections (3.9 cases per 100,000 population), and 14 cases of invasive SDSE infections (3.2 cases per 100,000 population; Table 2). When testing antibiotic susceptibility by the disc diffusion method [7], all SDSE isolates were sensitive to penicillin, whereas 6% of *S. anginosus* group isolates were resistant. Two SDSE isolates (14%) were resistant to clindamycin and one (7%) was resistant to erythromycin (Table 2).

**Table 2:** **Characteristics of invasive SDSE and *S. anginosus *group infections in a one-year period at London health sciences centre**

**Characteristics**	**SDSE**	***S. anginosus *group**
Patients, n	14	17
Rate (per 100,000 population)	3.2	3.9
Source of organism, n (%)		
Blood	11 (79)	6 (35)
Tissue	3 (21)	10 (59)
Cerebrospinal fluid (CSF)	0 (0)	1 (6)
Antibiotic resistance, n (%)		
Penicillin	0 (0)	1 (6)
Ceftriaxone	-	1 (6)
Erythromycin	1 (7)	2 (12)
Clindamycin	2 (14)	2 (12)
Vancomycin	-	0 (0)

## Conclusions

Fournier’s gangrene was first identified in 1883, when the French dermatologist and venereologist Jean Alfred Fournier diagnosed a rapidly progressive gangrene of the genitalia with no discernible etiology in five young men [13]. Now defined as a necrotizing fasciitis of the perineal or genital areas [1,2], Fournier’s gangrene remains unusually rare, with an incidence ranging from 0.002% to 0.005% of annual hospital admissions [14]. The infection can rapidly spread throughout the perineum, thighs, and torso, subsequently leading to gangrene, septic shock, and death if untreated. Although Group A *streptococci* were thought to be the sole cause of Fournier’s gangrene [15], subsequent clinical series have emphasized the polymicrobial nature of the disease [1,16], which is hypothesized to synergize enzyme production and promote rapid multiplication and spread of infection [1]. The most common causative microorganisms include facultative organisms (*E. coli*, *Klebsiella*, enterococci), along with anaerobes (*Bacteroides*, *Fusobacterium*, *Clostridium*, or anaerobic or microaerophilic *Streptococci*). This case is unique because the patient lacked the typical risk factors associated with Fournier’s gangrene, such as diabetes, immune compromise, obesity, drug use, or genital trauma [1-3], and his infection was caused by *Streptococcus dysgalactiae subsp. equisimilis* (SDSE).

SDSE, a pyogenic β-hemolytic streptococcus [17], usually colonizes the upper respiratory, gastrointestinal, and female genital tracts [17]. However, it is increasingly being recognized as an important human pathogen [18], with a wide spectrum of disease similar to that caused by *S. pyogenes*[5], including endocarditis, rheumatic fever, and streptococcal toxic shock-like syndrome [6]. In a recent population-based study, the burden of invasive SDSE infections approximated that of invasive *S. pyogenes* infections [4]. SDSE primarily presents as skin and soft-tissue infections, including pyoderma, cellulitis, wound infections, abscesses, erysipelas, and necrotizing fasciitis [4]. SDSE contains either Lancefield group antigens C or G, but needs to be distinguished from *S. anginosus* group strains, which frequently contain the same antigens. The use of MALDI-TOF MS to differentiate between streptococcal species has been established [19-21], although misidentification may occur because of striking similarities in proteomic profiles [22,23]. The organism in our case was misidentified as *S. anginosus* group by MALDI-TOF MS, because sequencing the 16S rRNA segment confirmed the isolate as SDSE. While few laboratories report identification of β-hemolytic Group C and G streptococci to the species level [24], differentiation should not be ignored because SDSE is more invasive than the *S. anginosus* group [25], and may have virulence factors similar to *S. pyogenes*[26,27]. Although the presence of streptococcal superantigens such as SpeG homologues have been described in SDSE [6,28], our clinical isolate lacked Group A streptococcal superantigen genes and activity. Nevertheless, our case demonstrates the potential benefit of molecular assays in differentiating closely-related streptococcal species, although further studies are needed to assess their clinical impact. To our knowledge, there are no other reports of Fournier’s gangrene caused by *Streptococcus dysgalactiae* subspecies *equisimils* (SDSE). One study isolated Group C *Streptococcus* from the perineum of a diabetic male with Fournier’s gangrene [29], but the species was not reported, and a potential role for superantigens was not investigated.

At our institution, the incidences of invasive *S. anginosus* group and SDSE infections were 3.9 and 3.2 cases per 100,000 respectively (Table 2). The rate of invasive *S. anginosus* group infection is slightly higher than previous studies [4], while the rate of invasive SDSE infection is slightly lower. Although rare penicillin-resistant SDSE strains have been reported [30], isolates from our centre were sensitive to penicillin. Six percent of *S. anginosus* group isolates, however, were resistant to penicillin, which is higher than other studies [31]. While the mechanisms of resistance have yet to be fully elucidated, the potential transfer of penicillin resistance determinants from related Streptococcal species [32], together with selective antibiotic pressure, may play a role in the emergence of penicillin resistance in the *S. anginosus* group [4,33]. Therefore, the addition of an aminoglycoside to a cell wall-active agent may be appropriate for severe *S. anginosus* group infections to avoid delayed response of infection [33]. Similar to other studies [30] showing widespread resistance to macrolides (16-24%) we also observed erythromycin resistance (12% and 7% for *S. anginosus* group and SDSE isolates, respectively), albeit at a lower rate. Additionally, 12% of *S. anginosus* group isolates and 14% of SDSE isolates were resistant to clindamycin, suggesting that despite the popularity of macrolide and clindamycin use in infected patients, they may not be appropriate for all cases.

In conclusion, we present a case of Fournier’s gangrene of the penis caused by SDSE, highlighting a unique disease presentation of the organism, and underscoring the limitations of MALDI-TOF MS in differentiating between closely-related streptococcal species which may hixave differing pathogenic profiles.

## Consent

Written informed consent was obtained from the patient for publication of this case report. A copy of the written consent is available for review by the editor of this journal.

## Competing interests

The authors report no actual or potential conflicts of interest.

## Authors’ contributions

RVA collected the tissue culture sample, contributed to the discussion of the results, and drafted and wrote the manuscript. KJK carried out the PCR amplification of the 16S rRNA and superantigen genes and contributed to the discussion of the results. KGP performed the proliferation assay and generated the supplementary figures. JJZ carried out the *emm* sequencing. JD and JKM contributed to the assessment and discussion of the results. All authors read and approved the final manuscript.

## Pre-publication history

The pre-publication history for this paper can be accessed here:

http://www.biomedcentral.com/1471-2334/13/381/prepub

## Supplementary Material

Additional file 1: Figure S1 PCR analysis of S. dysgalactiae subspecies equisimilis (SDSE) chromosomal DNA did not detect any known Group A streptococcal superantigen genes. S. dysgalactiae subspecies equisimilis (s), positive control (S. pyogenes MGAS5005, SF370, MGAS8232, and MGAS315; +), and negative control (no template). **Figure S2:** Supernatant from the clinical isolate identified as S. dysgalactiae subspecies equisimilis (SDSE) failed to induce the proliferation of human PBMCs. Human PBMCs were incubated with supernatant dilutions from S. pyogenes strain MGAS5005 or the clinical isolate for 72h and subsequently pulsed with [3H]thymidine to assess mitogenic activity. DNA was harvested after 18 h, and the counts per minute (cpm) were determined by scintillation counting and normalized. The mean (± SEM) of experiments performed in quadruplicate are shown. Click here for file
