# Supplementary material for: Fournier’s gangrene of the penis caused by Streptococcus dysgalactiae subspecies equisimilis: case report and incidence study in a tertiary-care hospital
Source: BMC Infect Dis. 2013 Aug 20;13:381. doi: 10.1186/1471-2334-13-381 (PMC3751815; doi:10.1186/1471-2334-13-381)
Supplement: Additional file 1: Figure S1 — PCR analysis of S. dysgalactiae subspecies equisimilis (SDSE) chromosomal DNA did not detect any known Group A streptococcal superantigen genes. S. dysgalactiae subspecies equisimilis (s), positive control (S. pyogenes MGAS5005, SF370, MGAS8232, and MGAS315; +), and negative control (no template). Figure S2: Supernatant from the clinical isolate identified as S. dysgalactiae subspecies equisimilis (SDSE) failed to induce the proliferation of human PBMCs. Human PBMCs were incubated with supernatant dilutions from S. pyogenes strain MGAS5005 or the clinical isolate for 72h and subsequently pulsed with [3H]thymidine to assess mitogenic activity. DNA was harvested after 18 h, and the counts per minute (cpm) were determined by scintillation counting and normalized. The mean (± SEM) of experiments performed in quadruplicate are shown. [file 1471-2334-13-381-S1.docx]

# Additional file

#
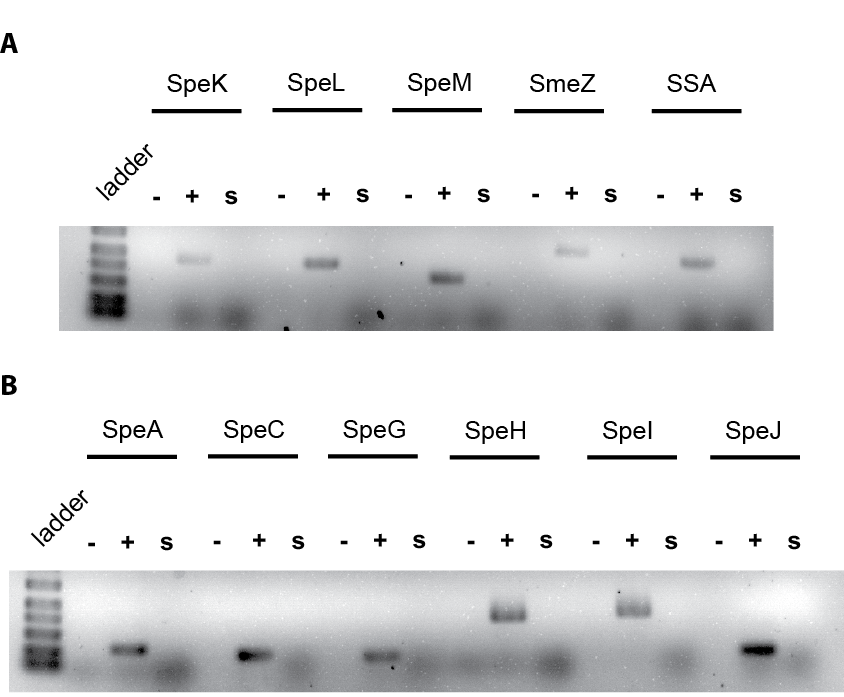


Figure 1: PCR analysis of *S. dysgalactiae* subspecies *equisimilis* (SDSE) chromosomal DNA did not detect any known Group A streptococcal superantigen genes. *S. dysgalactiae* subspecies *equisimilis* (s), positive control (*S. pyogenes* MGAS5005, SF370, MGAS8232, and MGAS315; +), and negative control (no template).

Figure 2: Supernatant from the clinical isolate identified as *S. dysgalactiae* subspecies *equisimilis* (SDSE) failed to induce the proliferation of human PBMCs. Human PBMCs were incubated with supernatant dilutions from *S. pyogenes* strain MGAS5005 or the clinical isolate for 72h and subsequently pulsed with [^3^H]thymidine to assess mitogenic activity. DNA was harvested after 18 h, and the counts per minute (cpm) were determined by scintillation counting and normalized. The mean (± SEM) of experiments performed in quadruplicate are shown.
